# Disentanglement—Induced Superconductivity

**DOI:** 10.3390/e27060630

**Published:** 2025-06-13

**Authors:** Eyal Buks

**Affiliations:** Andrew and Erna Viterbi Department of Electrical Engineering, Technion, Haifa 32000, Israel; eyal@ee.technion.ac.il

**Keywords:** disentanglement

## Abstract

The current study is motivated by a difficulty in reconciling between particle number conservation and superconductivity. An alternative modeling, which is based on the hypothesis that disentanglement spontaneously ocuurs in quantum systems, is explored. The Fermi–Hubbard mode is employed to demonstrate a disentanglement-induced quantum phase transition into a state with a finite superconducting order parameter. Moreover, the effect of disentanglement on Josephson junction’s current phase relation is explored

## 1. Introduction

In the Bardeen, Cooper, and Schrieffer (BCS) model [1], the Hamiltonian $H_{\mathrm{BCS}}$ of electrons in a superconducting metal contains interaction terms proportional to the operators $B_{k^{\prime \prime }}^{†}B_{k^{\prime }}$, where $B_{k^{\prime }}=a_{-k^{\prime },↓}a_{k^{\prime },↑}$ is a pair annihilation operators, and $a_{k^{\prime },\sigma }$ annihilates a single particle Fermionic state having momentum $\hbar k^{\prime }$ and spin state $\sigma \in \left\{↑,↓\right\}$. The operator $B_{k^{\prime \prime }}^{†}B_{k^{\prime }}$ can be expressed as $B_{k^{\prime \prime }}^{†}B_{k^{\prime }}=C_{k^{\prime },k^{\prime \prime }}+B_{k^{\prime \prime }}^{†}\langle B_{k^{\prime }}\rangle +\langle B_{k^{\prime \prime }}^{†}\rangle B_{k^{\prime }}-\langle B_{k^{\prime \prime }}^{†}\rangle \langle B_{k^{\prime }}\rangle$, where $\langle B_{k^{\prime }}\rangle$ is the expectation value of $B_{k^{\prime }}$ in thermal equilibrium, and $C_{k^{\prime },k^{\prime \prime }}=\left(B_{k^{\prime \prime }}^{†}-\langle B_{k^{\prime \prime }}^{†}\rangle \right)\left(B_{k^{\prime }}-\langle B_{k^{\prime }}\rangle \right)$. In the mean field approximation (MFA), the term $C_{k^{\prime },k^{\prime \prime }}$ is disregarded [see Equation (18.307) of Ref. [2]]. This approximation leads to a mean field Hamiltonian $H_{\mathrm{MF}}$, which can be analytically diagonalized by implementing a Bogoliubov transformation.

The MFA greatly simplifies the many-body problem under study; however, it yields some predictions that are arguably inconsistent with what is expected from the original Hamiltonian $H_{\mathrm{BCS}}$. Particle number is conserved by $H_{\mathrm{BCS}}$, and consequently, it is expected that in steady state, $\langle B_{k^{\prime }}\rangle =0$. In contrast, $\left|\langle B_{k^{\prime }}\rangle \right|$, which is proportional to the BCS energy gap, can become finite in the MFA. Moreover, the ground state of the mean-field Hamiltonian $H_{\mathrm{MF}}$ is continuously degenerate, whereas the ground state of the BCS Hamiltonian $H_{\mathrm{BCS}}$ is generically non-degenerate. The question of MFA validity is related to the spontaneous symmetry breaking in the Higgs mechanism [3].

It was pointed out that the MFA can be, at least partially, justified in the thermodynamical limit. Particle number conservation implies that $\langle N_{P}^{2}\rangle -{\langle N_{P}\rangle }^{2}=0$ in steady states, where $N_{P}=\left(1/2\right)\sum_{k^{\prime }}a_{k^{\prime },↑}^{†}a_{k^{\prime },↑}+a_{k^{\prime },↓}^{†}a_{k^{\prime },↓}$ is the pair number operator. In general, the MFA allows the violation of this conservation law (i.e., it allows non-zero values of $\langle N_{P}^{2}\rangle -{\langle N_{P}\rangle }^{2}$ in steady state). However, it was shown that in the MFA, both $\langle N_{P}\rangle$ and $\langle N_{P}^{2}\rangle -{\langle N_{P}\rangle }^{2}$ are proportional to the volume of the system [4], and thus, the violation of particle number conservation becomes insignificant in the thermodynamical limit. The mean field approach has been supported in Ref. [5] by showing that the Ginzburg–Levanyuk parameter is typically small for electrons in metals. Moreover, it was argued in Ref. [6] that the BCS interaction between pairs has an infinite range, and consequently exact solution of the BCS Hamiltonian $H_{\mathrm{BCS}}$ can be derived using a MFA. It was shown in Ref. [7] that the Bogoliubov inequality, together with a variational calculation and some assumptions, can lead to the MFA Hamiltonian $H_{\mathrm{MF}}$. Another attempt to rigorously derive the MFA Hamiltonian $H_{\mathrm{MF}}$, which is based on Wick’s theorem [8], has been presented in [9,10]. However, this derivation employs a relation, which can be derived from Wick’s theorem only for the case of Gaussian states [see Equation (16.131) of Ref. [2]]. In contrast, the thermal equilibrium state that is derived from the BCS Hamiltonian $H_{\mathrm{BCS}}$ is generically non-Gaussian.

The current study is motivated by the arguably limited range of validity of the MFA, and by the difficulty in reconciling between the spontaneous symmetry breaking occurring in the superconducting state, and particle number conservation [11,12,13]. An alternative approach, which is based on a recently proposed hypothesis that disentanglement spontaneously occurs in quantum systems, is explored. As is shown below, the conjecture that disentanglement plays a role in superconductivity is falsifiable, since it yields predictions that are distinguishable from what is derived from MFA-based models. In the current study, the Fermi–Hubbard model [14,15,16,17,18,19,20,21,22,23] is employed to study the effect of disentanglement on both superconducting order parameter and current-phase relation (CPR) of a weak link [24].

## 2. Disentanglement

According to the spontaneous disentanglement hypothesis, time evolution for the reduced density operator ρ is governed by a modified master equation given by [25,26,27,28,29]

(1) $$\frac{d\rho }{dt}=i\hbar^{-1}\left[\rho ,H\right]-\Theta \rho -\rho \Theta +2\langle \Theta \rangle \rho \;,$$

where *ℏ* is the Planck’s constant, $H=H^{†}$ is the Hamiltonian, the operator $\Theta =\Theta^{†}$ is allowed to depend on ρ, and $\langle \Theta \rangle =\mathrm{Tr}\left(\Theta \rho \right)$. The operator Θ is given by $\Theta =\gamma_{H}Q^{\left(H\right)}+\gamma_{D}Q^{\left(D\right)}$, where both rates $\gamma_{H}$ and $\gamma_{D}$ are positive, and both operators $Q^{\left(H\right)}$ and $Q^{\left(D\right)}$ are Hermitian. The operator $Q^{\left(H\right)}$, which gives rise to thermalization [30,31], is given by $Q^{\left(H\right)}=\beta U_{H}$, where $U_{H}=H+\beta^{-1}\log\rho$ is the Helmholtz free energy operator [32], $\beta =1/\left(k_{B}T\right)$ is the thermal energy inverse, $k_{B}$ is the Boltzmann’s constant, and *T* is the temperature.

For the case of a system composed of indistinguishable particles, the disentanglement operator $Q^{\left(D\right)}$ is derived from two–particle interaction (TPI) [33]. The term in the Hamiltonian H accounting for TPI is denoted by V. In a basis that diagonalizes the TPI, the operator V is expressed in terms of the operators $N_{j^{\prime }}N_{j^{\prime \prime }}$, where $N_{j}$ is a number operator associated with the *j*’th single-particle state. In that basis, each term in V proportional to $N_{j^{\prime }}N_{j^{\prime \prime }}$ contributes to $Q^{\left(D\right)}$, a term proportional to $Q_{j^{\prime },j^{\prime \prime }}\langle Q_{j^{\prime },j^{\prime \prime }}\rangle$, where $Q_{j^{\prime },j^{\prime \prime }}=N_{j^{\prime }}N_{j^{\prime \prime }}-\langle N_{j^{\prime }}\rangle \langle N_{j^{\prime \prime }}\rangle$. The term $Q_{j^{\prime },j^{\prime \prime }}\langle Q_{j^{\prime },j^{\prime \prime }}\rangle$ gives rise to suppression of $C_{j^{\prime },j^{\prime \prime }}^{2}$, with a rate proportional to $\gamma_{D}$, where the covariance $C_{j^{\prime },j^{\prime \prime }}$ is defined by $C_{j^{\prime },j^{\prime \prime }}=\langle \left(N_{j^{\prime }}-\langle N_{j^{\prime }}\rangle \right)\left(N_{j^{\prime \prime }}-\langle N_{j^{\prime \prime }}\rangle \right)\rangle =\langle Q_{j^{\prime },j^{\prime \prime }}\rangle$ [see Equation (1)]. Alternatively, the covariance $C_{j^{\prime },j^{\prime \prime }}$ can be expressed as $C_{j^{\prime },j^{\prime \prime }}=p_{j^{\prime },j^{\prime \prime }}-p_{j^{\prime }}p_{j^{\prime \prime }}$, where $p_{j}$ is the probability that state *j* is occupied, and $p_{j^{\prime },j^{\prime \prime }}$ is the probability that states $j^{\prime }$ and $j^{\prime \prime }$ are both occupied.

## 3. Fermi–Hubbard Model

Consider an array of sites occupied by Fermions. Single-site occupation energy, nearest neighbors hopping, and TPI are characterized by the real parameters μ, *t*, and *U*, respectively. The creation and annihilation operators corresponding to site *l* with spin state $\sigma \in \left\{↑,↓\right\}$ are denoted by $a_{l,\sigma }^{†}$ and $a_{l,\sigma }$, respectively. The operators $a_{l,\sigma }^{†}$ and $a_{l,\sigma }$ satisfy Fermionic anti-commutation relations. The Fermi–Hubbard Hamiltonian H is given by $H=H_{0}+V$, where the single-particle part $H_{0}$ is

(2) $$\begin{array}{cc} H_{0} & =-t\sum_{\sigma \in \left\{↑,↓\right\}}\sum_{\langle l^{\prime },l^{\prime \prime }\rangle }\left(a_{l^{\prime },\sigma }^{†}a_{l^{\prime \prime },\sigma }+a_{l^{\prime \prime },\sigma }^{†}a_{l^{\prime },\sigma }\right)\\ \; & -\mu \sum_{\sigma \in \left\{↑,↓\right\}}\sum_{l}a_{l,\sigma }^{†}a_{l,\sigma }\;, \end{array}$$

where $\langle l^{\prime },l^{\prime \prime }\rangle$ denotes that $l^{\prime }$ and $l^{\prime \prime }$ are nearest neighbors, the TPI part is given by

(3) $$V=U\sum_{l}\left(N_{l,↑}-\frac{1}{2}\right)\left(N_{l,↓}-\frac{1}{2}\right)\;,$$

and the Fermionic number operator $N_{l,\sigma }$ is given by $N_{l,\sigma }=a_{l,\sigma }^{†}a_{l,\sigma }$.

The term $N_{l,↑}N_{l,↓}$ in the TPI part V [see Equation (3)] can be expressed as $N_{l,↑}N_{l,↓}=C_{l}+N_{l,↑}\langle N_{l,↓}\rangle +\langle N_{l,↑}\rangle N_{l,↓}-\langle N_{l,↑}\rangle \langle N_{l,↓}\rangle$, where $C_{l}=\left(N_{l,↑}-\langle N_{l,↑}\rangle \right)\left(N_{l,↓}-\langle N_{l,↓}\rangle \right)$. In the MFA, i.e., when the term $C_{l}$ is disregarded, it is well known that the Fermi–Hubbard model supports a superconducting phase for particular realizations [34].

As was discussed above, disentanglement gives rise to the suppression of the covariance $\langle C_{l}\rangle$. In the rapid disentanglement approximation [35], it is assumed that the rate of disentanglement $\gamma_{D}$ is sufficiently large to allow disregarding the term $C_{l}$. In this limit, the disentanglement-based model yields predictions that are identical to what is derived from the standard (i.e., without disentanglement) Fermi–Hubbard model, when the MFA is implemented, and thus, the disentanglement-based model in this limit can account for superconductivity, in the same way that the mean field Fermi–Hubbard model can.

In the current study, the effect of disentanglement is explored, without assuming that $\gamma_{D}$ is sufficiently large to validate the rapid disentanglement approximation. As is demonstrated below, for some cases, analytical results can be derived from the modified master Equation (1), provided that the size of the under study system is kept sufficiently small. However, since the rapid disentanglement approximation is not implemented, analysis commonly becomes intractable in the macroscopic limit.

For the relatively simple systems to be discussed below, it is assumed that the Fermi–Hubbard array is one-dimensional; the number of sites, which is denoted by *L*, is finite; and the array has a ring configuration; thus, the last (l=L) hopping term $a_{l,\sigma }^{†}a_{l+1,\sigma }+a_{l+1,\sigma }^{†}a_{l,\sigma }$ [see Equation (2)] is taken to be given by $a_{L,\sigma }^{†}a_{1,\sigma }+a_{1,\sigma }^{†}a_{L,\sigma }$.

## 4. Truncation Approximation

For some cases, dynamics governed by the modified master Equation (1) can be simplified by implementing a truncation approximation. In this approximation, the operators H and Θ are replaced by PHP and PΘP, respectively, where *P* is a projection operator. For a two-level truncation approximation, the projection P is expressed as $P=|\psi_{1}\rangle \langle \psi_{1}|+|\psi_{2}\rangle \langle \psi_{2}|$, where $|\psi_{1}\rangle$ and $|\psi_{2}\rangle$ are two orthonormal state vectors (i.e., $\langle \psi_{1}|\psi_{1}\rangle =\langle \psi_{2}|\psi_{2}\rangle =1$ and $\langle \psi_{1}|\psi_{2}\rangle =0$). The density operator ρ for that case is expressed in terms of the real vector $k=\left(k_{x},k_{y},k_{z}\right)$ as

(4) $$\rho \dot{=}\frac{1+\sigma \cdot k}{2}\;,$$

where $\sigma =\left(\sigma_{x},\sigma_{y},\sigma_{z}\right)$ is the Pauli matrix vector. Similarly, the Hamiltonian is expressed as $\hbar^{-1}H\dot{=}\sigma \cdot \omega$, where $\omega =\left(\omega_{x},\omega_{y},\omega_{z}\right)$ is real. It is assumed that $Q^{\left(D\right)}=Q\langle Q\rangle$, where $Q\dot{=}q_{0}+q\cdot \sigma$, and both the number $q_{0}$ and the vector $q=\left(q_{x},q_{y},q_{z}\right)$ are real.

The entropy operator −logρ can be expressed as $-\log\rho \dot{=}-\log\sqrt{\left(1-k^{2}\right)/4}-\left(\tanh^{-1}k\right)\sigma \cdot \hat{k}$, where $k=\left|k\right|$ and $\hat{k}=k/k$, and the operator Θ as $\Theta =s_{0}+\sigma \cdot s$, where $s_{0}=\gamma_{H}\langle \log\rho \rangle +\gamma_{D}q_{0}\langle Q\rangle$, $s=\gamma_{H}\beta \hbar \omega +\gamma_{D}\langle Q\rangle q$, and $\langle Q\rangle =q_{0}+q\cdot k$ [recall the identity $\left(\sigma \cdot u\right)\left(\sigma \cdot v\right)=u\cdot v+i\sigma \cdot \left(u\times v\right)$, and note that the Pauli matrices are all trace-less]. The modified master Equation (1) yields an equation of motion for k, given by

(5) $$\frac{dk}{dt}=-2\left(k\times \omega +s-\left(s\cdot k\right)k\right)\;.$$

Note that, generally, s depends on k, and that the vector $s-\left(s\cdot k\right)k$ is orthogonal to k, provided that k=1 (i.e., ρ represents a pure state, for which $\mathrm{Tr}\rho^{2}=1$).

When the Hamiltonian H is time-independent, steady-state solutions of the modified master Equation (1) occur at extremum points of an effective free energy $\langle U_{e}\rangle$, which is given by $\langle U_{e}\rangle =\gamma_{H}^{-1}\beta^{-1}\langle \Theta \rangle =\langle U_{H}\rangle +\beta^{-1}\left(\gamma_{D}/\gamma_{H}\right)\langle Q^{\left(D\right)}\rangle$. In the truncation approximation, $\beta \langle U_{H}\rangle =\beta \hbar \omega \cdot k+\langle \log\rho \rangle$, where

(6) $$\langle \log\rho \rangle =\frac{1-k}{2}\log\frac{1-k}{2}+\frac{1+k}{2}\log\frac{1+k}{2}\;,$$

and $\langle Q^{\left(D\right)}\rangle ={\langle Q\rangle }^{2}={\left(q_{0}+q\cdot k\right)}^{2}$. For a constant ω, the Helmholtz free energy $\langle U_{H}\rangle$ is minimized at the thermal equilibrium point $k=-\tanh\left(\beta \hbar \omega \right)\hat{\omega }$, where the unit vector $\hat{\omega }$ is given by $\hat{\omega }=\omega /\left|\omega \right|$ [note that $d\langle \log\rho \rangle /dk=\tanh^{-1}k$].

For the under-study Fermi–Hubbard model, and for the case of a two-site array (i.e., L=2) and μ=0, a two-level truncation approximation, which is based on a projection onto the subspace spanned by the floor $|f\rangle$ (i.e., ground) and ceiling $|c\rangle$ energy eigenstates, becomes applicable, provided that $\left|t/U\right|\ll 1$ [33]. For the case μ=0, the floor $|f\rangle$ and ceiling $|c\rangle$ states are given by $|f\rangle =\cos\left(\alpha \right)|X\rangle +\sin\left(\alpha \right)|Y\rangle$ and $|c\rangle =\sin\left(\alpha \right)|X\rangle -\cos\left(\alpha \right)|Y\rangle$, where $|X\rangle =2^{-1/2}\left(|0011\rangle +|1100\rangle \right)$, $|Y\rangle =2^{-1/2}\left(|0110\rangle +|1001\rangle \right)$, $\alpha =\left(1/2\right)\tan^{-1}\left(-8t/U\right)$, and $|\eta_{4}\eta_{3}\eta_{2}\eta_{1}\rangle$ denotes a normalized state, where $\eta_{1}=\langle N_{1,↑}\rangle \in \left\{0,1\right\}$, $\eta_{2}=\langle N_{1,↓}\rangle \in \left\{0,1\right\}$, $\eta_{3}=\langle N_{2,↑}\rangle \in \left\{0,1\right\}$ and $\eta_{4}=\langle N_{2,↓}\rangle \in \left\{0,1\right\}$. Note that the disentanglement expectation value $\langle Q^{\left(D\right)}\rangle$ with respect to the state $|\vartheta \rangle \equiv \cos\left(\vartheta \right)|X\rangle +\sin\left(\vartheta \right)|Y\rangle$, where the angle ϑ is real, is given by $\langle Q^{\left(D\right)}\rangle =\left(\gamma_{D}/8\right)\cos^{2}\left(2\vartheta \right)$. Hence, in the limit $\left|t/U\right|\ll 1$, for which $|f\rangle ≃|X\rangle$ and $|c\rangle ≃-|Y\rangle$, the combined state $2^{-1/2}\left(|f\rangle -|c\rangle \right)≃|\vartheta =\pi /4\rangle$ is nearly fully disentangled.

**Figure 1:**
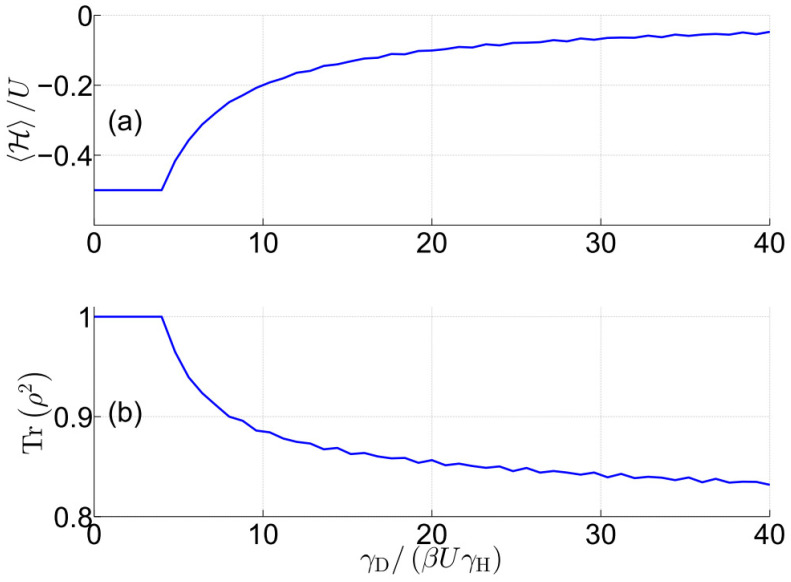
Fermi–Hubbard model. Steady-state values of (**a**) normalized energy expectation value $\langle H\rangle /U$ and (**b**) purity $\mathrm{Tr}\left(\rho^{2}\right)$ as a function of the ratio $\gamma_{D}/\left(\beta U\gamma_{H}\right)$. A symmetry-breaking quantum phase transition occurs at $\gamma_{D}/\left(\beta U\gamma_{H}\right)=4$. Assumed parameters’ values are $t/U=10^{-3}$ and μ=0.

The relations $\hbar \omega =E_{0}\left(0,0,1\right)$, $q_{0}=0$ and $q=\left(-t/E_{0},0,U/\left(8E_{0}\right)\right)$, where $E_{0}=\left(1/2\right)\sqrt{U^{2}+64t^{2}}$, enables an analytical evaluation of the effective free energy $\langle U_{e}\rangle$. The result reveals that in the low-temperature limit, and for $\left|t/U\right|\ll 1$, a symmetry-breaking quantum phase transition occurs for this case at $\gamma_{D}/\left(\beta U\gamma_{H}\right)=4$. The dependency on the ratio $\gamma_{D}/\left(\beta U\gamma_{H}\right)$ of steady-state values of (a) the normalized energy expectation value $\langle H\rangle /U$ and (b) purity $\mathrm{Tr}\left(\rho^{2}\right)$ is shown in Figure 1. The steady-state values are calculated by numerically integrating the modified master Equation (1) (without employing the truncation approximation). The plot in Figure 1b reveals that the purity $\mathrm{Tr}\left(\rho^{2}\right)$ drops below unity above the phase transition occurring at $\gamma_{D}/\left(\beta U\gamma_{H}\right)=4$.

## 5. Order Parameter

**Figure 2:**
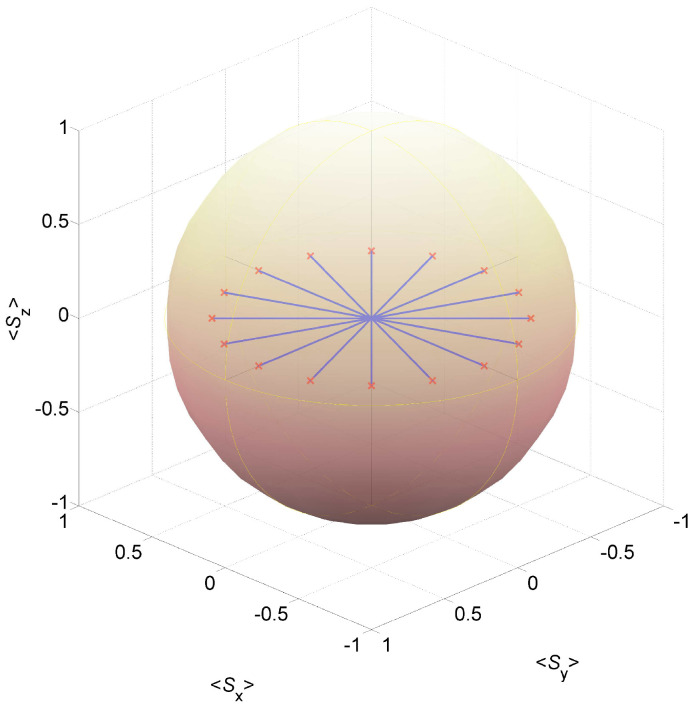
Disentanglement-induced spontaneous symmetry breaking for the case L=2. Time evolution of the vector $\langle S\rangle$ for different initial states located close to the ground state $|f\rangle \langle f|$ [for which $\langle S\rangle =\left(0,0,0\right)$]. The assumed parameters’ values are $\epsilon_{s}=10^{-4}$, t/U=0.01, μ/U=0, and $\gamma_{D}/\left(\beta U\gamma_{H}\right)=50$.

The plot in Figure 2 demonstrates the time evolution of the vector $\langle S\rangle =\left(\langle S_{x}\rangle ,\langle S_{y}\rangle ,\langle S_{z}\rangle \right)$ for the case L=2 [the truncation approximation is not being employed for the numerical integration of the modified master Equation (1)]. The vector operator S is given by $S=\sum_{l=1}^{L}S_{l}$, where $S_{l}=\left(S_{l,x},S_{l,y},S_{l,z}\right)=\Theta_{l}^{†}\sigma \Theta_{l}$, and where $\Theta_{l}^{†}=\left(\begin{array}{cc} a_{l,↑}^{†}, & a_{l,↓} \end{array}\right)$. The following holds ${\left[S_{l^{\prime },i},S_{l^{\prime \prime },j}\right]}_{-}=2i\epsilon_{ijk}\delta_{l^{\prime },l^{\prime \prime }}S_{l^{\prime },k}$, $S_{l,+}\equiv S_{l,x}+iS_{l,y}=2B_{l}^{†}$, $S_{l,-}\equiv S_{l,x}-iS_{l,y}=2B_{l}$ and $S_{l,z}=-1+N_{l}$, where $B_{l}=a_{l,↓}a_{l,↑}$ and where $N_{l}=N_{l,↑}+N_{l,↓}$, and thus $S_{l^{\prime }}\cdot S_{l^{\prime \prime }}=2\left(B_{l^{\prime }}^{†}B_{l^{\prime \prime }}+B_{l^{\prime \prime }}^{†}B_{l^{\prime }}\right)+2\left(1-N_{l^{\prime }}\right)\delta_{l^{\prime },l^{\prime \prime }}+\left(1-N_{l^{\prime }}\right)\left(1-N_{l^{\prime \prime }}\right)$ (note that $B_{l}^{†}B_{l}=a_{l,↑}^{†}a_{l,↓}^{†}a_{l,↓}a_{l,↑}=N_{l,↑}N_{l,↓}$). The variable ${\langle S_{x}\rangle }^{2}+{\langle S_{y}\rangle }^{2}$ represents an order parameter.

In the low-temperature limit, and in the absence of disentanglement (i.e., for $\gamma_{D}=0$), the ground state density operator $|f\rangle \langle f|$ is a steady-state solution of the modified master Equation (1). Note that $\langle S\rangle =\left(0,0,0\right)$ for the ground state $|f\rangle \langle f|$. Above the disentanglement-induced quantum phase transition, i.e., for $\gamma_{D}/\left(\beta U\gamma_{H}\right)>4$, the ground state becomes unstable. For the assumed parameters’ values used to generate the plot in Figure 2, the ratio $\gamma_{D}/\left(\beta U\gamma_{H}\right)$ is 50 (see figure caption). The plot shows time evolution for 16 different initial pure states, denoted by $\rho_{i}\left(\theta_{s}\right)=|\psi_{i}\rangle \langle \psi_{i}|/\langle \psi_{i}|\psi_{i}\rangle$, where $|\psi_{i}\rangle$ is given by $|\psi_{i}\rangle =|f\rangle +\epsilon_{s}\left(|0011\rangle +e^{-i\theta_{s}}|1100\rangle \right)$, where $\epsilon_{s}\ll 1$ [i.e., $\rho_{i}\left(\theta_{s}\right)≃|f\rangle \langle f|$]. Time evolution, which is obtained by numerically integrating the modified master Equation (1), is shown for 16 equally spaced values for the angle $\theta_{s}$ in the range $\left[0,2\pi \right)$. The plot demonstrates that the steady-state value of $\langle S\rangle$ (labelled in Figure 2 by red × symbols) that is obtained with the initial state $\rho_{i}\left(\theta_{s}\right)$ is parallel to the unit vector $\left(\cos\theta_{s},\sin\theta_{s},0\right)$. Thus, for this one-dimensional model, a disentanglement-induced spontaneous symmetry breaking, which occurs for $\gamma_{D}/\left(\beta U\gamma_{H}\right)>4$, gives rise to finite values of the order parameter ${\langle S_{x}\rangle }^{2}+{\langle S_{y}\rangle }^{2}$.

## 6. CPR

For the case where the one-dimensional array is occupied by *spinless* Fermions, the Hamiltonian H is expressed as

(7) $$\begin{array}{cc} H & =\sum_{l=1}^{L}\left[-t_{l}\left(e^{i\varphi_{l}}a_{l}^{†}a_{l+1}+e^{-i\varphi_{l}}a_{l+1}^{†}a_{l}\right)+g_{l}B_{l}^{†}B_{l}\right]\\ \; & -\mu \sum_{l=1}^{L}\left(a_{l}^{†}a_{l}-\frac{1}{2}\right)\;. \end{array}$$

The Fermionic creation and annihilation operators corresponding to site $l\in \left\{1,2,\ldots ,L\right\}$ are denoted by $a_{l}^{†}$ and $a_{l}$, respectively, and the operator $B_{l}$ is given by $B_{l}=a_{l+1}a_{l}$ and $B_{L}=a_{1}a_{L}$. It is assumed that $t_{l}=t_{0}\delta_{l,L}+t\left(1-\delta_{l,L}\right)$ and $g_{l}=g_{0}\delta_{l,L}+g\left(1-\delta_{l,L}\right)$ (i.e., all nearest neighbor site pairs, except for the pair $\left(L,1\right)$, share the same coefficients, $t_{l}$ and $g_{l}$). The single-site occupation energy μ, hopping amplitudes *t* and $t_{0}$, the phases $\varphi_{l}$, and the pairing amplitudes *g* and $g_{0}$ are all real constants. For the case of an opened chain, $t_{0}=0$ and $g_{0}=0$, whereas $t_{0}=t$ and $g_{0}=g$ for the case of a closed ring.

The term $B_{l}^{†}B_{l}$ can be expressed as $B_{l}^{†}B_{l}=C_{l}+\langle B_{l}\rangle B_{l}^{†}+\langle B_{l}^{†}\rangle B_{l}-\langle B_{l}^{†}\rangle \langle B_{l}\rangle$, where $C_{l}=\left(B_{l}^{†}-\langle B_{l}^{†}\rangle \right)\left(B_{l}-\langle B_{l}\rangle \right)$. In the MFA, for which the term $C_{l}$ is disregarded, the resultant Hamiltonian, which is denoted by $H_{K}$, describes a Kitaev one-dimensional array [36]. Note that the total number of particles is conserved by H [see Equation (7)], whereas only the total number mod 2 is conserved by $H_{K}$. In the analysis below, the MFA, which generally enables violation of number conservation, is not implemented.

Consider the case where a magnetic flux given by $\phi_{e}=\nu \phi_{0}$ is externally applied to the ring’s hole, where ν is real and $\phi_{0}=hc/e$ is the flux quantum (Planck’s constant, vacuum speed of light, and electronic charge are denoted by *h*, *c*, and *e*, respectively). The effect of the applied flux is taken into account by setting the phases $\varphi_{l}$ in the Hamiltonian (7) according to $\varphi_{l}=0$ for $l\in \left\{1,2,\ldots ,L-1\right\}$ and $\varphi_{L}=2\pi \nu$ [37,38]. The circulating current $\langle I\rangle$ is calculated using the relation $\langle I\rangle =-c\partial \langle H\rangle /\partial \phi_{e}$ [see Equation (18.142) of Ref. [2]], where the steady-state energy expectation value $\langle H\rangle$ is evaluated by numerically integrating the modified master Equation (1). For the current case, the disentanglement operator $Q^{\left(D\right)}$ is given by $Q^{\left(D\right)}=g_{0}Q_{L,1}\langle Q_{L,1}\rangle +g\sum_{l=1}^{L-1}Q_{l,l+1}\langle Q_{l,l+1}\rangle$, where $Q_{l^{\prime },l^{\prime \prime }}=N_{l^{\prime }}N_{l^{\prime \prime }}-\langle N_{l^{\prime }}\rangle \langle N_{l^{\prime \prime }}\rangle$ (note that $B_{l}^{†}B_{l}=N_{l}N_{l+1}$ and $B_{L}^{†}B_{L}=N_{L}N_{1}$, where $N_{l}=a_{l}^{†}a_{l}$).

**Figure 3:**
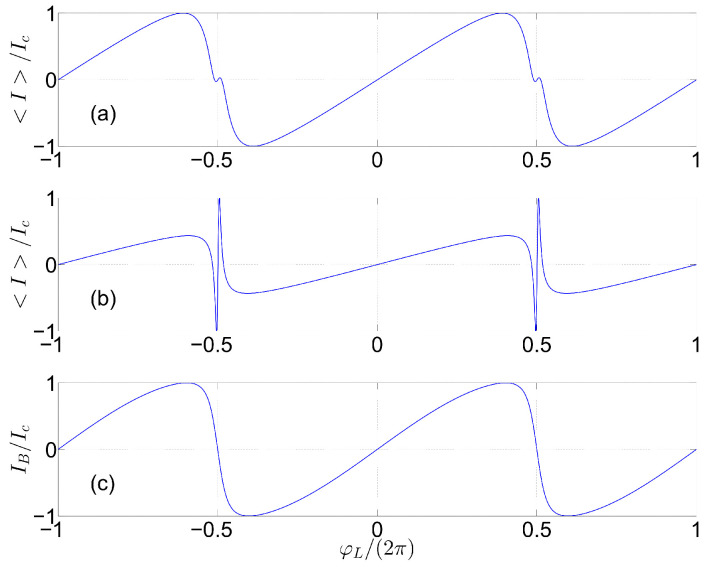
CPR: The normalized circulating current $\langle I\rangle /I_{c}$ is shown as a function of normalized applied flux $\varphi_{L}/\left(2\pi \right)=\nu$, where $I_{c}$ is the critical current. The assumed parameters’ values are, L=5, g/t=1, $t_{0}/t=0.8$, $g_{0}/t=0$, and μ/t=0, for (**a**,**b**); $\gamma_{D}/\gamma_{H}=5$, for (**a**); $\gamma_{D}/\gamma_{H}=10$, for (**b**); and τ=0.99 for (**c**).

The effect of disentanglement on CPR is demonstrated by the plots shown in Figure 3. The assumed rate of disentanglement $\gamma_{D}$ for the plots in (a) and (b) is $\gamma_{D}/\gamma_{H}=5$, and $\gamma_{D}/\gamma_{H}=10$, respectively. For comparison, the plot in Figure 3c displays the Beenakker–VanHouten CPR $I_{B}\left(\varphi_{L}\right)$ [39,40], which was calculated for a single short channel of transmission τ, and which is given by $I_{B}\left(\varphi_{L}\right)=I_{c}F\left(\varphi_{L}\right)$, where $I_{c}$ denotes the critical current, and [see Equation (A4) of Ref. [41]]

(8) $$F\left(\varphi_{L}\right)=\frac{\tau \sin\varphi_{L}}{2\sqrt{2\left(1-\sqrt{1-\tau }\right)-\tau }\sqrt{1-\tau \sin^{2}\left(\varphi_{L}/2\right)}}\;.$$

The most pronounced effect of disentanglement on the CPR are the sharp features seen in Figure 3a,b near half-integer values of the normalized applied flux $\varphi_{L}/\left(2\pi \right)$. These features do not violate the symmetry relation $I\left(\varphi_{L}/\left(2\pi \right)-n-1/2+x\right)=-I\left(\varphi_{L}/\left(2\pi \right)-n-1/2-x\right)$, where *n* is an integer. Note that some unexplained features obeying the same symmetry are visible in some spectral measurements of Josephson devices (e.g., see Figures 2 and 4 of [42], Figure 2 of [43], and Figure 2 of [44]). Further study is needed to determine whether disentanglement can account for such experimentally observed features. Note that a variety of unconventional mechanisms, including topological and multi-band superconductivity, can give rise to CPR having features that resemble what is seen in Figure 3a,b (e.g., see Ref. [45]).

## 7. Effective Free Energy

**Figure 4:**
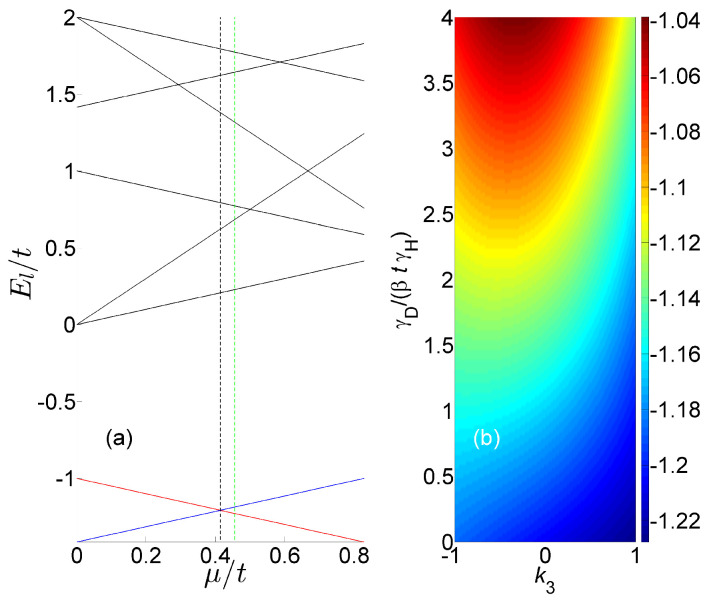
Effective free energy. Chain parameters are L=3, g/t=1, $\varphi_{l}=0$ and $t_{0}=g_{0}=0$. (**a**) The energy eigenvalues $E_{l}$ of H (7). (**b**) The steady-state expectation value $\langle U_{e}\rangle /t$.

Disentanglement is explored below by evaluating the effective free energy $\langle U_{e}\rangle$ for the spinless one-dimensional array in an open chain configuration. The energy eigenvalues $E_{l}$ of H [see Equation (7)] are shown as a function of μ in Figure 4a, for the case where L=3, g/t=1, $\varphi_{l}=0$ and $t_{0}=g_{0}=0$. For $\mu <\mu_{c}$, where $\mu_{c}=\left(\sqrt{2}-1\right)t$ [see the black dashed vertical line in Figure 4a], the ground state is the one-particle state $|\psi_{1}\rangle =2^{-1}|100\rangle +2^{-1}|001\rangle +2^{-1/2}|010\rangle$ [see the blue line in Figure 4a], whereas the two-particle state $|\psi_{2}\rangle =6^{-1/2}|110\rangle +6^{-1/2}|011\rangle +2\times 6^{-1/2}|101\rangle$ [see the red line in Figure 4a] becomes the ground state for $\mu >\mu_{c}$.

Consider a reduced-density operator ρ having matrix representation in the basis $\left\{|\psi_{1}\rangle ,|\psi_{2}\rangle \right\}$ given by $\rho \dot{=}\left(1/2\right)\left(1+k\cdot \sigma \right)$, where $k=\left(k_{1},k_{2},k_{3}\right)$ is real. The truncated density operator ρ can be used for approximately calculating the effective free energy $\langle U_{e}\rangle$ for $\mu ≃\mu_{c}$. The dependency of $\langle U_{e}\rangle$ on $k_{3}$ and $\gamma_{D}/\left(\beta t\gamma_{H}\right)$ for the value $\mu /\mu_{c}=1.1$ [see the green dashed vertical line in Figure 4a] is shown in Figure 4b (note that $\langle U_{e}\rangle$ does not depend on $k_{1}$ and on $k_{2}$ in the truncation approximation). The color-coded plot of $\langle U_{e}\rangle$ reveals a disentanglement-induced transition from monostability to bistability. In the low-temperature limit, and in the absence of disentanglement [i.e., in the limit $\gamma_{D}/\left(\beta t\gamma_{H}\right)\to 0$], the effective free energy $\langle U_{e}\rangle$ is minimized for the two-particle state $|\psi_{2}\rangle$. However, for $\gamma_{D}≳\beta t\gamma_{H}$, the system becomes bistable [see Figure 4b].

## 8. Summary

Spontaneous disentanglement allows the violation of particle number conservation, which, in turn, enables a quantum phase transition induced by symmetry breaking. The Hubbard–Fermi model is employed for studying the effect of disentanglement on the superconducting order parameter and on the CPR of a weak link. While the current study is focused on exploring the effect of disentanglement on small systems, future research will explore the macroscopic limit using stability analysis [46] (this research direction has been proposed by one of the reviewers of this paper). Moreover, more realistic theoretical models that can yield experimentally testable predictions will be developed.

## Data Availability

Data are contained within the article.
